# Cardiovascular mortality trends and disparities in U.S. breast cancer patients, 1999–2020: a population-based retrospective study

**DOI:** 10.1186/s40959-024-00286-2

**Published:** 2024-12-19

**Authors:** Yong-Hao Yeo, Boon-Jian San, Jia-Yi Tan, Min-Choon Tan, Teodora Donisan, Justin Z. Lee, Laura M. Franey, Salim S. Hayek

**Affiliations:** 1 Department of Internal Medicine/ Pediatrics, Corewell Health William Beaumont University Hospital, Royal Oak, MI USA; 2 Department of Internal Medicine, Jacobi Medical Center, Albert Einstein College of Medicine, Bronx, NY USA; 3 Department of Internal Medicine, New York Medical College at Saint Michael’s Medical Center, Newark, NJ USA; 4 Department of Cardiovascular Medicine, Mayo Clinic, Phoenix, AZ USA; 5 Department of Cardiovascular Medicine, Mayo Clinic, Rochester, MN USA; 6 Department of Cardiovascular Medicine, Cleveland Clinic, Cleveland, OH USA; 7 Department of Cardiovascular Medicine, Corewell Health Grand Rapids, Michigan State University, Grand Rapids, MI USA; 8 Department of Cardiovascular Medicine, University of Texas Medical Branch, Galveston, TX USA; 9 Division of Cardiology, Department of Internal Medicine, University of Michigan, Ann Arbor, MI USA

## Abstract

**Background:**

Breast cancer survivors face a higher risk of cardiovascular disease (CVD) compared to non-breast cancer patients, yet contemporary data on CVD-related mortality within this group remains scarce.

**Objective:**

To investigate trends and disparities in CVD mortality among breast cancer patients.

**Methods:**

We queried the Centers for Disease Control and Prevention’s Wide-Ranging Online Data for Epidemiologic Research (CDC Wonder) and conducted serial cross-sectional analyses on national death certificate data for CVD mortality in breast cancer patients aged 25 and above from 1999 to 2020. We calculated age-adjusted mortality rates (AAMR) per 100,000 individuals and analyzed trends over time using the Joinpoint Regression Program, with further analyses stratified by age, race, census region, and urbanization level.

**Results:**

A total of 74,733 CVDs with comorbid breast cancer in the United States were identified between 1999 and 2020. The AAMR from CVDs with comorbid breast cancer decreased from 2.57 (95% CI [2.50–2.65]) in 1999 to 1.20 (95% CI [1.15–1.24]) in 2020, with an average annual percent change (AAPC) of − 4.3. The three most common causes of CVDs were ischemic heart disease (47.8%), cerebrovascular disease (17.1%), and hypertensive disease (10.6%). Our analysis revealed a significant decrease in AAMR for all CVD subtypes, except for hypertensive diseases and arrhythmias. The decrease in annual percent change (APC) was more pronounced in individuals aged ≥ 65 years compared to those < 65 years (-4.4, 95%CI [-4.9, -3.9] vs. -2.9, 95%CI [-4.1, -1.7], respectively. Notably, non-Hispanic Blacks consistently exhibited the highest AAMR (1.95, 95%CI [1.90–1.99]), whereas Hispanic or Latina patients had the lowest AAMR (0.75, 95% CI [0.72–0.78]). The AAMR was also higher in rural regions than in urban areas (1.64, 95%CI [1.62–1.67] vs. 1.55, 95%CI [1.53–1.56]).

**Conclusion:**

The study highlights a significant decline in CVD mortality among breast cancer patients over two decades, with persistent disparities by race and region. Exceptionally, hypertensive diseases and arrhythmias did not follow this declining trend.

**Supplementary Information:**

The online version contains supplementary material available at 10.1186/s40959-024-00286-2.

## Clinical perspective

**What is new?:**

There was a significant decline in CVD mortality among breast cancer patients from 1999 to 2020. However, deaths from hypertensive diseases and arrhythmias did not follow this declining trend. Non-Hispanic Black patients consistently had higher age-adjusted mortality rates from CVD than non-Hispanic White and Hispanic or Latina patients, showing ongoing racial disparities. Rural areas also had higher cardiovascular mortality rates than urban areas, indicating geographical disparities in health outcomes.

**What are the clinical implications?:**

While there was an improvement in population-level CVD mortality in patients with comorbid breast cancer, efforts are essential to address racial and geographical disparities.

## Introduction

In recent years, breast cancer has surpassed lung cancer as the most commonly diagnosed cancer globally [1]. With the improvement in breast cancer therapy, the mortality rate of breast cancer has declined by 1.3% per year from 2011 to 2017 [2], resulting in a growing number of breast cancer survivors. In the United States (U.S.) alone, there were over 4 million female breast cancer survivors in 2022 [3]. The longer life expectancy among patients with a history of breast cancer signifies the care for these patients extends beyond the cancer itself. A recent study showed that two-thirds of breast cancer patients died of non-cancer causes, with cardiovascular deaths being the most prevalent, constituting 30.6%[4]. The high risk of cardiovascular disease (CVD) in breast cancer patients may stem from various factors, including shared risk factors for both conditions (such as obesity, hypertension, and diabetes mellitus), the cardiotoxic effects of breast cancer therapies, and the pro-inflammatory and pro-thrombotic state associated with breast cancer itself [5–7].

Previous studies have sought to examine the trends of CVD among breast cancer patients [8, 9]. However, these studies lacked comprehensive nationwide coverage and did not include analysis of various subtypes of CVDs. In addition, the increased vigilance in screening for cardiotoxicity and the establishment of Cardio-Oncology services over the past decade warrant a re-examination of trends in cardiovascular outcomes in patients with breast cancer [10, 11]. There has also been a growing recognition of how social determinants—including economic stability, neighborhood and social cohesion, food security, education, and healthcare access—could impact cardiovascular health in cancer survivors [12]. We sought to leverage nationwide data using the Centers for Disease Control and Prevention’s Wide-Ranging Online Data for Epidemiologic Research (CDC WONDER) database to assess the trends in mortality attributed to CVDs with comorbid breast cancer, including the mortality trends for each subtype of CVD. Understanding changes in these trends may yield insights into changes in population-level mortality related to CVD, in addition to race and geographical disparities.

## Methods

### Data source

We conducted a retrospective cross-sectional study to determine the trends in CVD mortality among patients with comorbid breast cancer from 1999 to 2020. The data was obtained from the Multiple Cause of Death Database in the CDC WONDER. The Multiple Cause of Death database includes the underlying and contributing causes of death from all death certificates in the U.S. International Classification of Diseases, Tenth Revision (ICD-10) is used to classify the causes of death for the years 1999 to 2020. Each death certificate includes one underlying cause of death and up to 20 contributing causes of death. The World Health Organization defines the underlying cause of death as the disease or injury that initiates a sequence of events that leads directly to death [13]. All deaths occurring in hospitals and out-of-hospital settings among U.S. residents were captured, while deaths of nonresidents were excluded. No prior institutional review approval was required as the data is deidentified and publicly available.

### Definitions

Using the CDC WONDER database, diseases of the circulatory system (ICD-10 I00-I99) were listed as the underlying causes of death, and breast cancer (ICD-10 C50) was listed as the contributing cause of death. Patients with comorbid breast cancer are defined as the individuals in which breast cancer was listed as the contributing cause of death in the death certificate. Individuals with unknown causes of death stated on the death certificates at the time of death were excluded. Further analyses were performed for each CVD subtypes such as hypertensive diseases (I10–I15), ischemic heart disease (I20–I25), pulmonary heart disease (I26–I28), valvular heart diseases (I05-I09, I34-I37), cardiomyopathy(I42), heart failure (I50), arrhythmia (I44-I49), cerebrovascular disease (I60–I69), peripheral vascular diseases (I70-78, I80-89) and other cardiovascular death (Pericardial Diseases [I30-I32], Endocarditis [I33, I38], Myocarditis [I40], Ill-Defined Heart Diseases [I51]). This study methodology has been validated in other similar research topics [14, 15].

### Study outcomes

First, we calculated the age-adjusted mortality rates (AAMRs) per 100,000 individuals using the direct method by applying age-specific rates in a population of interest to 2000 U.S. Standard Population [16]. This reduces the confounding effects of varying age structures and enables meaningful comparisons across different populations. We plotted the AAMR per 100,000 individuals to determine the trends from the year 1999 to 2020. The trend of proportionate mortality was determined by dividing the number of cardiovascular deaths among patients with comorbid breast cancer by the number of all-cause mortality deaths among patients with comorbid breast cancer. We also extracted data on age, race, and geographical regions to compare age-adjusted mortality rates (AAMRs) from a demographic perspective. Age was categorized in 10-year intervals starting from 25 years, as no deaths were recorded in individuals aged 24 years and below. Race and ethnicity were classified as Hispanic or Latina, non-Hispanic White, and non-Hispanic Black. For geographical variations, we cross-examined the AAMRs across different regions in the U.S. and the degrees of urbanization [17]. The population was further categorized into urban (large central metro, large fringe metro, medium metro, and small metro counties) and rural (micropolitan non-metro and non-core non-metro counties) according to the 2013 U.S. Census Classifications [18, 19]. Additionally, we included AAMRs at the state level for all 50 states and the District of Columbia.

### Statistical analysis

We used Joinpoint Regression Program (Joinpoint V4.9.1.0, National Cancer Institute) to evaluate trends of AAMRs in each subgroup. This method, as described in previous similar studies, determines the significance of AAMR changes over time using log-linear regression models where temporal variation occurred [20, 21]. Annual Percent Change (APC) with 95% confidence intervals (CI) for the AAMRs was calculated using the Monte Carlo permutation test at the identified line segments linking joinpoint. Afterward, the weighted averages of the APCs, also known as average annual percent change (AAPC) were calculated with corresponding 95% CI, which reflects the summary of the mortality trends in the study period.

## Results

Among the 4,473,854,489 individuals aged 25 and above in our study period from the year 1999 to 2020, there were a total of 1,125,693 deaths from all-cause mortality among patients with comorbid breast cancer, of which 74,733 deaths (6.6%) were related to CVDs. The majority of them were females (73,770, 98.7%). Further exploration revealed that 10,148 deaths (13.6%) had a concomitant diagnosis of diabetes mellitus, 3,557 deaths (4.8%) had hyperlipidemia, 3,569 deaths (4.8%) had nicotine dependence, and 723 deaths (0.97%) had obesity (Table 1).

**Table 1:** Baseline characteristics of all-cause Mortality and Cardiovascular Death among patients with comorbid breast Cancer

Demographic	All-Cause, *n*(%)	CVD Related to Breast Cancer, *n*(%)
	*n* = 1,125,693	*N* = 74,733
**Sex**		
Female	1,113,442 (98.91)	73,770 (98.71)
**Age of death**, **year**		
25–34	8,757 (0.78)	43 (0.06)
35–44	50,561 (4.49)	345 (0.46)
45–54	132,589 (11.78)	1,540 (2.06)
55–64	207,980 (18.48)	4,433 (5.93)
65–74	236,749 (21.03)	10,204 (13.65)
75–84	260,115 (23.11)	22,283 (29.82)
85+	228,942 (20.34)	35,885 (48.02)
**Race and Ethnicity**		
Hispanic or Latina	59,783 (5.31)	2,439 (3.26)
Non-Hispanic Black	151,911 (13.49)	7,944 (10.63)
Non-Hispanic White	882,702 (78.41)	62,762 (83.98)
**Census Region**		
Region 1	227,021 (20.17)	17,352 (23.22)
Region 2	263,253 (23.39)	18,528 (24.79)
Region 3	405,610 (36.03)	22,675 (30.34)
Region 4	229,809 (20.41)	16,178 (21.65)
**Concomitant Diagnoses** **(Major Risk Factors for CVDs)**		
Diabetes Mellitus	65,960 (5.86)	10,148 (13.58)
Hyperlipidemia	16,434 (1.46)	3,557 (4.76)
Nicotine Dependence	37,980 (3.37)	3,569 (4.78)
Obesity	3,929 (0.35)	723 (0.97)

**Fig. 1:**
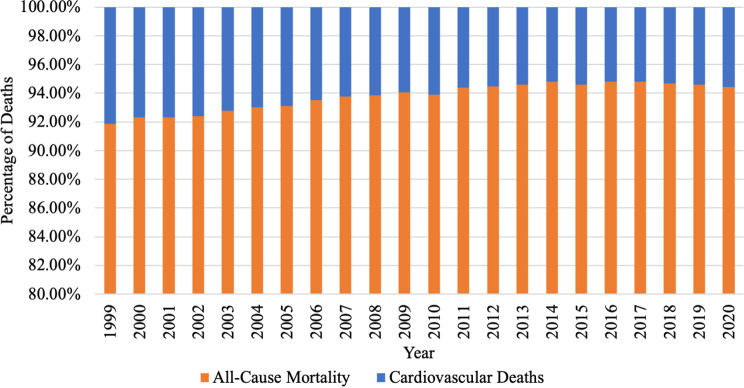
Trends in Number of Deaths of All-Cause Mortality vs. Cardiovascular Death among Patients with Comorbid Breast Cancer between 1999 and 2020

**Fig. 2:**
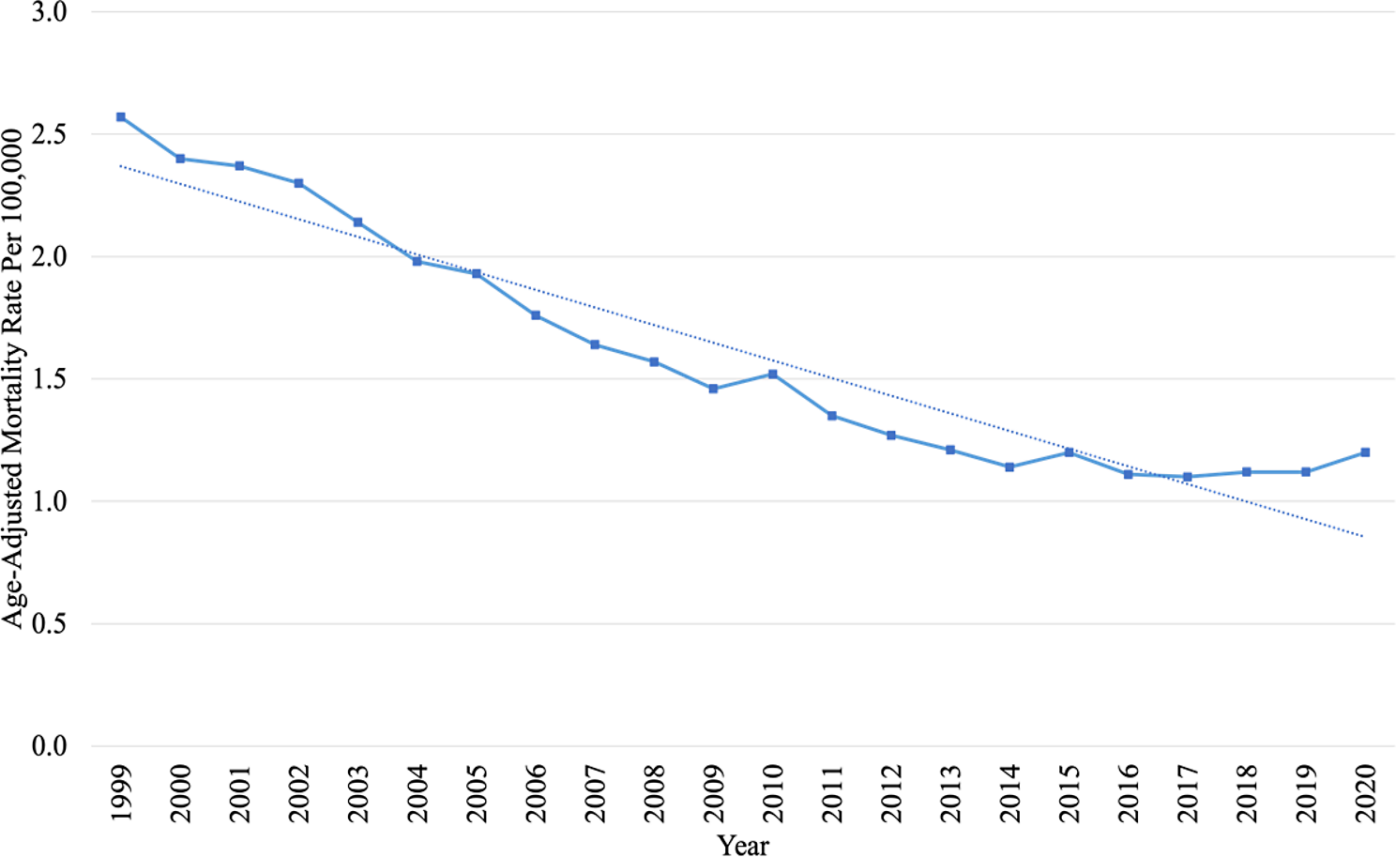
Trends in Age-Adjusted Mortality Rates of Cardiovascular Death among Patients with Comorbid Breast Cancer between 1999 and 2020. *The dashed line represents the trendline

**Fig. 3:**
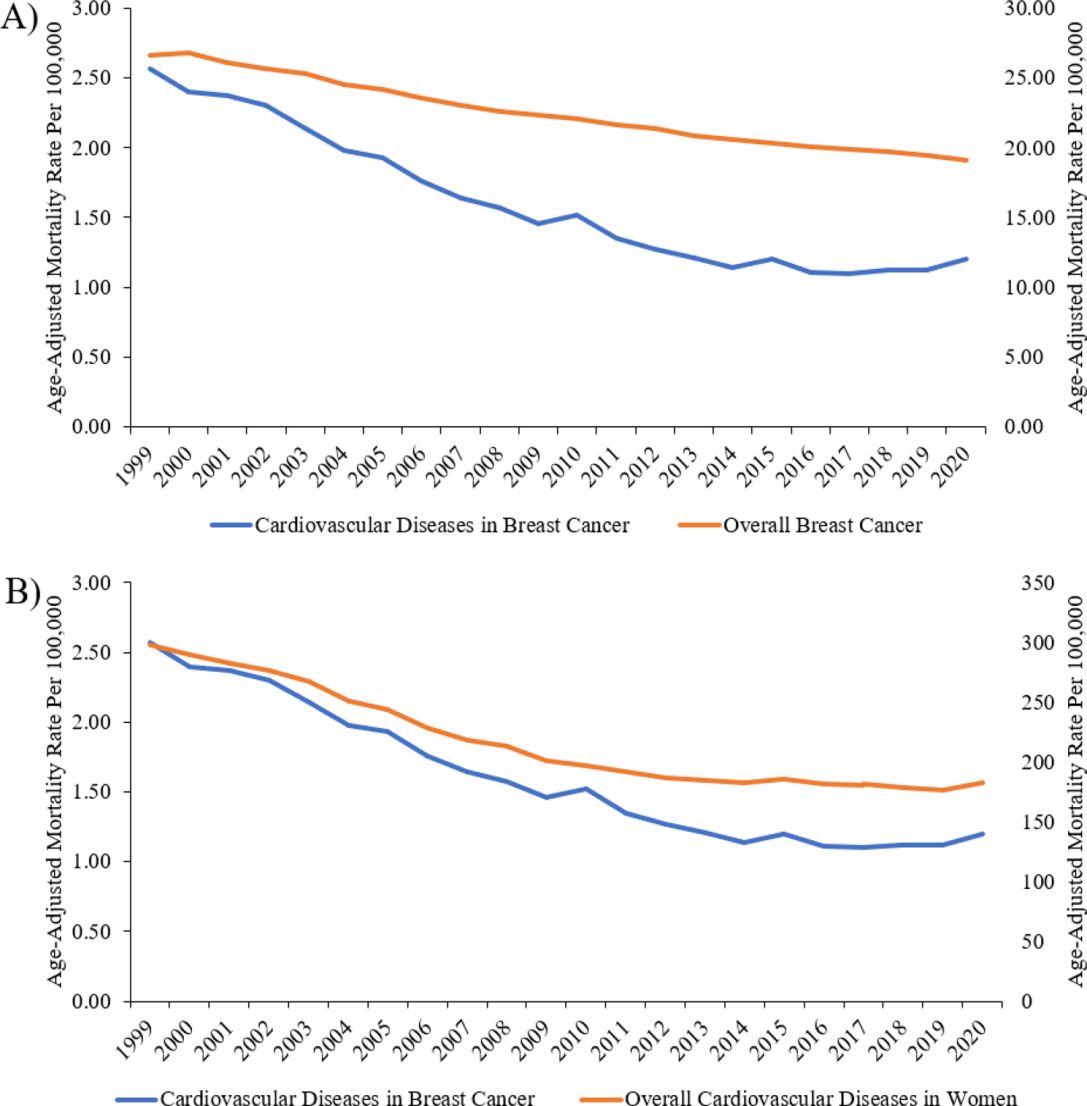
Comparison of Trends of Age-Adjusted Mortality Rate of CVDs in Breast Cancer vs. **A**. Age-Adjusted Mortality Rate of Overall Breast Cancer. **B**. Age-Adjusted Mortality Rate of Overall CVDs in Women

Proportionate mortality of CVDs decreased from 8.8% in 1999 to 5.5% in 2016, followed by an increase to 5.9% in 2020 (Fig. 1). The AAMR of CVDs among patients with comorbid breast cancer decreased from 2.57 (95% CI, 2.50–2.65) per 100,000 individuals in 1999 to 1.20 (95% CI, 1.15–1.24) per 100,000 individuals in 2020, with an average annual percent of change (AAPC) of − 4.3 (95% CI, -4.8, -3.8) (Fig. 2). An inflection point is noted in 2014, where the decrease in AAPC was significant before this at -5.4 (95% CI, -5.8, -4,9) but insignificant after this at − 0.1 (95% CI, -1.7, 1.6). The reduction in mortality rate for CVDs among patients with comorbid breast cancer was higher than the overall decrease observed in breast cancer mortality and overall CVD deaths in women over the same study period (AAPC − 4.3 vs. -1.7 vs. -2.6) (Fig. 3A and B).

### Subtypes of CVDs among patients with breast cancer

**Fig. 4:**
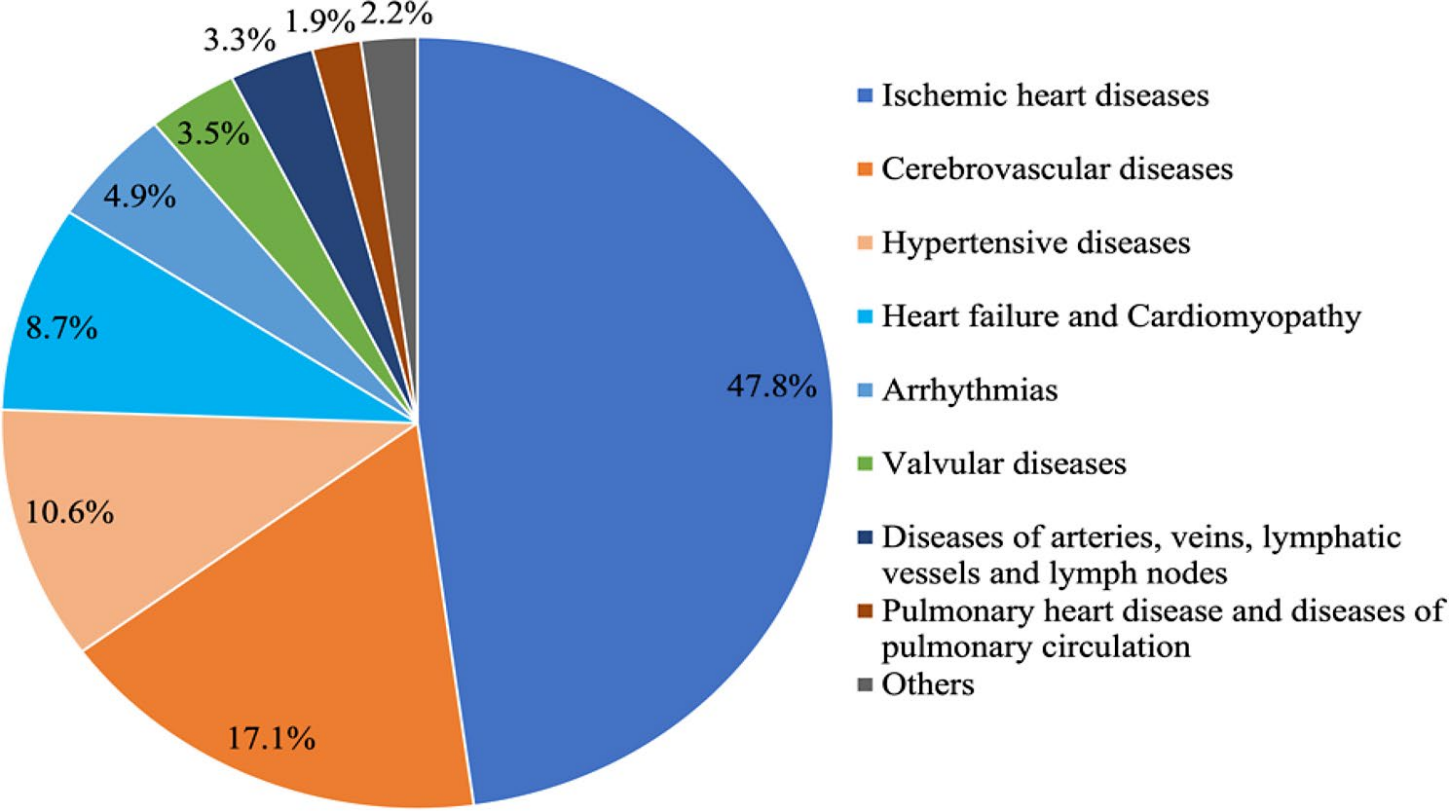
Etiologies of Cardiovascular Deaths among Patients with Comorbid Breast Cancer

Figure 4 depicts the etiologies of CVDs among patients with comorbid breast cancer. The most common cause was ischemic heart disease (47.8%), followed by cerebrovascular disease (17.1%), hypertensive disease (10.6%), congestive heart failure and cardiomyopathy (8.7%), arrhythmias (4.9%), valvular diseases (3.5%), diseases of arteries, veins, lymphatic vessels and lymph nodes (3.3%), pulmonary heart disease and diseases of pulmonary circulation (1.9%), and others (2.2%). AAMR trend analysis across 22 years (Table 2) revealed significant decrement in all subtypes of CVDs, except for hypertensive diseases (0.15 in 1999 [95% CI, 0.13–0.17] to 0.19 in 2020 [95% CI, 0.17–0.20]), and arrhythmias (0.08 in 1999 [95% CI, 0.06–0.09] to 0.08 in 2020 [95% CI, 0.07–0.09]). The decrease in AAPC was the greatest in diseases of arteries, veins, lymphatic vessels, and lymph nodes (-9.0 [95% CI, -10.8, -7.2] and least in hypertensive diseases (-0.1, [95% CI, -0.7, 0.6]). The only subtype of CVD with a positive AAPC was arrhythmia (0.4, [95% CI, -0.4, 1.2]).

**Table 2:** Trends in Age-Adjusted Mortality Rates of Cardiovascular Death among patients with comorbid breast Cancer between 1999 and 2020, stratified by subtypes of CVDs

			AAMR																
**Year**	**Population**, **No.**		**Ischemic Heart Diseases**		**Cerebrovascular Diseases**		**Hypertensive Diseases**		**Heart Failure and Cardiomyopathy**		**Arrhythmias**		**Valvular Heart Diseases**		**Diseases of Arteries**,** Veins and Lymphatic**		**Pulmonary Heart Diseases**		**Others**
1999	180,408,769		1.39		0.45		0.15		0.20		0.08		0.06		0.10		0.05		0.04
2000	181,984,640		1.28		0.44		0.18		0.19		0.07		0.06		0.10		0.06		0.03
2001	184,305,128		1.26		0.4		0.16		0.21		0.07		0.08		0.10		0.05		0.03
2002	186,208,028		1.2		0.36		0.17		0.19		0.08		0.06		0.09		0.05		0.03
2003	188,090,429		1.07		0.37		0.17		0.18		0.06		0.05		0.07		0.05		0.03
2004	190,205,384		1.01		0.35		0.15		0.17		0.06		0.06		0.08		0.04		0.02
2005	192,551,384		0.98		0.31		0.17		0.16		0.06		0.06		0.07		0.03		0.04
2006	195,019,359		0.9		0.28		0.17		0.17		0.08		0.05		0.05		0.01		0.04
2007	197,403,777		0.84		0.28		0.16		0.12		0.07		0.05		0.05		0.01		0.03
2008	199,795,090		0.81		0.26		0.16		0.11		0.09		0.06		0.04		0.02		0.03
2009	202,107,016		0.75		0.24		0.16		0.12		0.07		0.05		0.03		0.02		0.03
2010	203,891,983		0.75		0.25		0.16		0.12		0.07		0.06		0.03		0.01		0.02
2011	206,592,936		0.65		0.22		0.16		0.11		0.07		0.05		0.03		0.01		0.03
2012	208,826,037		0.61		0.23		0.14		0.11		0.06		0.05		0.04		0.01		0.03
2013	211,085,314		0.57		0.21		0.13		0.11		0.07		0.05		0.02		0.01		0.02
2014	213,809,280		0.51		0.19		0.15		0.09		0.07		0.05		0.02		0.01		0.02
2015	216,553,817		0.48		0.2		0.17		0.11		0.07		0.05		0.02		0.02		0.03
2016	218,641,417		0.46		0.18		0.15		0.12		0.07		0.04		0.01		0.01		0.03
2017	221,447,331		0.44		0.2		0.15		0.09		0.08		0.04		0.02		0.01		0.03
2018	223,311,190		0.45		0.2		0.16		0.09		0.08		0.04		0.02		0.02		0.02
2019	224,981,167		0.44		0.19		0.18		0.09		0.08		0.04		0.03		0.01		0.03
2020	226,635,013		0.46		0.24		0.19		0.10		0.08		0.04		0.02		0.02		0.03
Total	4,473,854,489		1.39		0.45		0.15		0.20		0.08		0.06		0.10		0.05		0.04
**Percentage of total CVDs (%)**		47.82		17.08		10.63		8.65		4.90		3.53		3.29		1.89		2.20	
**Annual Percentage of Change (%)** **(95% CI)**		-5.84 (-6.22, -5.45)		-4.05 (-4.80, -3.29)		-0.05 (-0.67, 0.58)		-4.04 (-4.81, -3.27)		0.42 (-0.37, 1.22)		-2.29 (-2.99, -1.58)		-8.99 (-10.79, -7.15)		-7.30 (-10.45, -4.03)		-1.12 (-2.58, 0.36)	

Age-adjusted mortality rate per 100,000 person-years, directly standardized to the 2000 US Census population. *Abbreviations* AAMR, age-adjusted mortality rate

### Disparities in age group

When stratified by different age groups (younger group < 65 years old, older group ≥ 65 years old), the AAMR of CVDs decreased in both age groups (Supplemental Fig. S1). Among patients < 65 years old, the AAMR decreased from 0.19 (95% CI, 0.16–0.21) per 100,000 individuals in 1999 to 0.14 (95% CI, 0.12–0.15) per 100,000 individuals in 2020 with AAPC of − 2.9 (95% CI, -4.1, -1.7) (Supplemental Fig. S1A). AAMR among patients ≥ 65 years old also showed a similar decrease from 12.39 (95% CI, 12.02–12.77) per 100,000 individuals in 1999 to 5.56 (95% CI, 5.36–5.77) per 100,000 individuals in 2020 with a greater AAPC of − 4.4 (95% CI, -4.9, -3.9) (Supplemental Fig. S1B).

### Racial and ethnicity differences

**Fig. 5:**
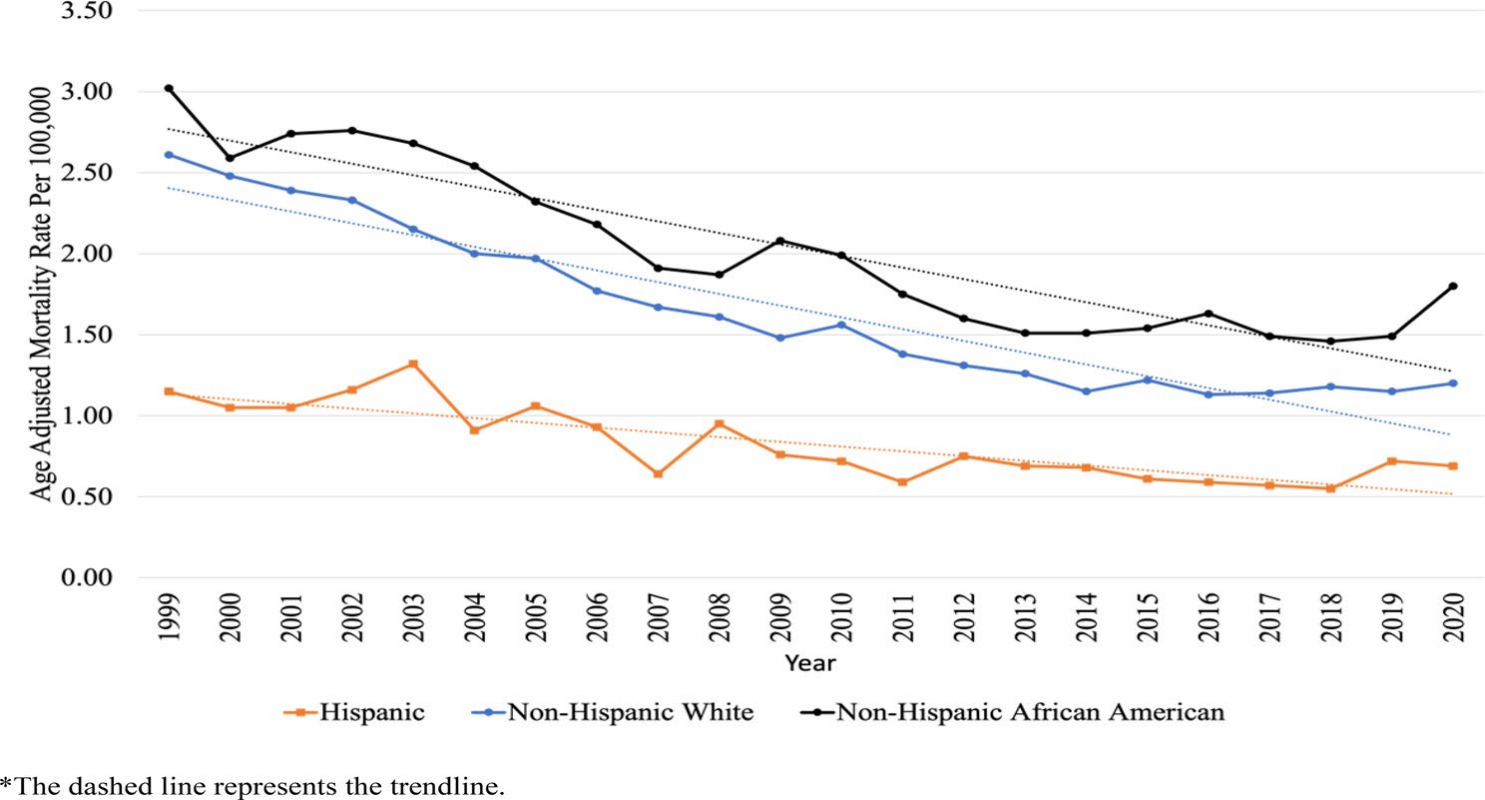
Trends in Age-Adjusted Mortality Rates of Cardiovascular Death among Patients with Comorbid Breast Cancer between 1999 and 2020, Stratified by Races and Ethnicities. *The dashed line represents the trendline

Throughout the study period, non-Hispanic Black patients with comorbid breast cancer consistently recorded the highest AAMR in CVDs with an average of 1.95 (95% CI, 1.90–1.99) per 100,000 individuals (Fig. 5). Hispanic or Latina patients with comorbid breast cancer had the lowest AAMR with an average of 0.75 (95% CI, 0.72–0.78) per 100,000 individuals throughout the years. The non-Hispanic Black patients also had the least decrease in AAPC (non-Hispanic Blacks − 3.4 [95% CI, -4.0, -2.7] vs. non-Hispanic White − 4.2 [95% CI, -4.7, -3.7] vs. Hispanic or Latina − 3.4 [95% CI − 4.4, -2.4]).

### Regional and urbanization differences

The AAMR in CVDs among patients with comorbid breast cancer was the highest in the Northeast regions (1.79 per 100,000 individuals [95% CI 1.76–1.82]), followed by the Midwest regions (1.68 per 100,000 individuals [95% CI 1.66–1.71]), West regions (1.62 per 100,000 individuals [95% CI 1.59–1.64]) and South regions (1.33 per 100,000 individuals [95% CI 1.31–1.34]). The median AAMRs for all States was 1.52 per 100,000 individuals (Supplemental Fig. S2). Among the five states with the highest AAMR, three were from the Midwest regions: North Dakota (2.19 per 100,000 individuals [95% CI, 1.93–2.44]), Ohio (2.15 per 100,000 individuals [95% CI, 2.09–2.22]), and Nebraska (2.09 per 100,000 individuals [95% CI, 1.93–2.25]), while two were from the South regions: District of Columbia (2.66 per 100,000 individuals [95% CI, 2.32–3.01]) and Oklahoma (2.20 per 100,000 individuals [95% CI, 2.07–2.32]). For the five states with the lowest AAMR, three were from the West regions: Nevada (0.72 per 100,000 individuals [95% CI, 0.63–0.82]), Arizona (0.95 per 100,000 individuals [95% CI, 0.89–1.01]), Utah (1.04 per 100,000 individuals [95% CI, 0.92–1.16]) and two were from South regions: Florida (0.88 per 100,000 individuals [95% CI, 0.85–0.91] and Georgia (1.04 per 100,000 individuals [95% CI, 0.98–1.10]).

In terms of urbanization, the AAMR in CVDs among patients with comorbid breast cancer was higher in rural regions compared to urban areas (1.64 per 100,000 individuals [95% CI, 1.62–1.67] vs. 1.55 per 100,000 individuals [95% CI, 1.53–1.56]). Trend analysis demonstrated that both urban and rural regions showed a decline in AAMRs across the study period. The decrease in AAPC was less in rural areas than in urban areas (–3.6 [95% CI, -4.1, -3.0] vs. − 4.4 [95% CI, -4.9, -3.9]).

## Discussion

We conducted a comprehensive analysis of 22 years of Centers for Disease Control and Prevention’s mortality data to examine the patterns of CVD mortality among patients with comorbid breast cancer. Our results reveal several important aspects: First, the major causes of cardiovascular deaths in this population are ischemic heart disease, cerebrovascular disease, hypertensive disease, congestive heart failure, cardiomyopathy, and arrhythmias. Second, we observed a positive trend of lower cardiovascular deaths as a proportion of all causes and lower AAMR from CVDs in patients with comorbid breast cancer. This trend may reflect the advances made in managing cardiovascular risks in this group. Third, despite overall improvements, non-Hispanic Black patients consistently had higher AAMR from CVDs than non-Hispanic White and Hispanic or Latina patients, showing ongoing disparities. Lastly, our analysis showed that rural areas had higher cardiovascular mortality rates than urban areas, indicating geographical disparities in health outcomes.

Cardiovascular mortality constituted 6.6% of all-cause mortality among patients with comorbid breast cancer. Meanwhile, 86.0% of all-cause mortality was attributed to breast cancer itself. This aligned with previous studies, which showed that CVDs attributed to 3.6 − 15.9% of deaths among breast cancer patients [22, 23]. The common causes were ischemic heart diseases, cerebrovascular disease, hypertensive disease, congestive heart failure and cardiomyopathy, and arrhythmias. Our studies indicated favorable outcomes were not seen in all the CVD subtypes. Deaths from hypertensive disease exhibited no significant decrement in AAMR, while the AAPC of AAMR for deaths from arrhythmias was positive. The increase in AAMR from arrhythmias is particularly interesting given that cardiomyopathy, a common cause of arrhythmias in patients with breast cancer, demonstrated a decreasing trend in our study. This underscores the need for developing more targeted cardioprotective strategies to mitigate hypertensive and arrhythmic risks both before and after cancer treatment.

Our study revealed some optimistic findings: the trends of both proportionate mortality of CVD (deaths from CVDs divided by all-cause mortality) and AAMRs of CVDs among patients with comorbid breast cancer decreased throughout the study period. Our findings were consistent with the existing study using the Surveillance, Epidemiology, and End Results (SEER) program, which included data from the 1970s to 2010 [24]. This improvement in mortality trend could be attributed to multiple reasons. Firstly, the decrease in the CVDs mortality trend could be due to better survival and a declining mortality rate in breast cancer and CVDs among women in general (Supplementary Figs. S1 and S2) [25, 26]. Secondly, multiple guidelines and consensus statements were released in the 2010s focusing on cardiac screening among patients with breast cancers. These recommendations advocated for utilizing multifaceted cardiac surveillance tests, such as echocardiogram and B-type natriuretic peptide (BNP), for early detection of cardiac dysfunction among breast cancer patients who received anthracycline therapy [27–30]. Thirdly, there has been a sharp decrease in anthracycline-based chemotherapy, with increased use of taxane-based chemotherapy for breast cancer since 2005 [31]. Lastly, the increased recognition of cardiotoxicity among breast cancer patients receiving therapy had led to the introduction of multiple cardioprotective strategies. The US Food and Drug Administration (FDA) approved dexrazoxane for cardioprotection in 1995 [32]. The American Society for Radiation Oncology guideline published in 2018 suggested methods to reduce radiation-induced cardiotoxicity, such as excluding the heart from the primary treatment field and using a deep inspiration breath-holding technique, which increases the distance between irradiated chest and heart [33, 34].

While these strategies hold promise, it is essential to note that most were introduced post-2010, except dexrazoxane usage. This may imply that the observed improvement in the AAMR of CVDs during our study’s initial phase may not be entirely attributed to recent innovations. In fact, our study showed an increase in proportionate mortality since 2016, and the AAPC of AAMR was not significant after 2014. Recognizing that the recent strides in cardioprotective strategies will require time for the advancements to be translated into improving mortality, future large-scale population-based studies evaluating the mortality benefits of these interventions are warranted.

When analyzing the disparities in the trend, our study found that non-Hispanic Black patients with comorbid breast cancer consistently recorded the highest AAMR of CVDs throughout the study period. Moreover, although there was a decrease in AAMRs in both races, the APC was smaller among Blacks than the White patients. These findings align with existing studies that have investigated the racial disparities in mortality rates for both breast cancer and CVD separately. While the incidence rate of breast cancer was lower among Blacks, they had a higher mortality rate than White patients [35]. Black female patients also constantly experienced higher cardiovascular mortality rates than their respective White counterparts [36]. These disparities may relate to differences in the prevalence of CV risk factors. Obesity had risen among Black women from 31% in the 1970s to 56% in the 2010s compared to White women from 15 to 39% in the same period [35]. Hypertension, another prevalent risk factor for CVDs, is also more frequently observed in the Black population, affecting approximately 45% of Blacks, compared to rates of 30–32% among non-Hispanic whites and Hispanics [37]. This finding might explain the non-significant decrease in hypertensive disease mortality seen in our analysis.

Social and economic factors could exacerbate this racial disparity. A study by Satti et al. showed that unfavorable social determinants of health profiles were associated with worse cardiovascular health outcomes among adult cancer survivors, particularly among women [38]. Multiple studies have shown that Black females faced more significant barriers to accessing timely high-level health care [36, 39, 40]. Sánchez-Díaz et al. found that neighborhood archetypes significantly impacted the cardiovascular health of Black breast cancer survivors [41]. Economic factors such as lack of insurance and poverty could be contributory [42]. Within the healthcare environment, they also encountered elevated levels of discrimination and racism [36, 39, 43]. Despite the introduction of new therapeutic agents, their utilization within this racial group has been sluggish, leading to a delayed decline in mortality trends [36, 44]. These factors contributing to challenging access to quality healthcare, resulting in racial disparities in mortality trends, may also account for the observed disparities between rural and urban regions [45, 46]. Targeted public health policies are essential to address the racial and regional disparities in AAMR of CVDs among patients with comorbid breast cancer. The recent scientific statement from the American Heart Association, which advocates for comprehensive strategies to reduce disparities in cardio-oncology care, is a commendable step forward [12]. However, translating these recommendations into tangible improvements in patient outcomes requires multilevel efforts, including local and public health interventions, as well as active engagement from clinicians to ensure effective implementation across all levels of care.

### Limitations

Our study has several major limitations. First, using ICD-10 codes alone as filter criteria imposes another limitation where there is no data regarding comorbidities, duration of cancer, staging, and treatment received, which directly affects the cardiovascular outcome. The temporal relationship between CVD and breast cancer cannot be ascertained as well. Second, the CDC-WONDER database does not consist of any information at individual levels where the pre-existing CV risk factors, socioeconomic factors, and distance to the nearest healthcare access can be important confounding factors in terms of cardiovascular mortality. Thirdly, the results relied heavily on accurate coding and determination of the causes of death. Finally, the lack of an option to choose the denominator for AAMR calculation, such as per 100,000 breast cancer patients instead of per 100,000 in the general population, could lead to an underestimation of the absolute rate of cardiovascular deaths among breast cancer patients. Despite these limitations, our study’s methodology and sample size allow the estimation of demographic and temporal relationships of CVDs with comorbid breast cancer among the U.S. population, and our study methodology has been validated in other similar research topics [47, 48].

## Conclusion

Our study revealed a decreasing trend in AAMR from CVDs with comorbid breast cancer during the 22-year study period. Further analysis showed that the observed improvement in mortality did not extend to deaths attributed to hypertensive diseases and arrhythmias. Our study also highlighted the racial and regional disparities in CVDs among patients with comorbid breast cancers.

## Electronic supplementary material

Below is the link to the electronic supplementary material.

Supplementary Material 1

## Data Availability

The data was obtained from the Multiple Cause of Death Database in the CDC WONDER. https://wonder.cdc.gov/mcd.html.
