# Supplementary material for: Cardiovascular mortality trends and disparities in U.S. breast cancer patients, 1999–2020: a population-based retrospective study
Source: Cardiooncology. 2024 Dec 19;10:89. doi: 10.1186/s40959-024-00286-2 (PMC11658079; doi:10.1186/s40959-024-00286-2)

Supplementary Figure 1. Trends in Age‐Adjusted Mortality Rates of Cardiovascular Death among Patients with Comorbid Breast Cancer between 1999 and 2020, Stratified by Age Group. A: < 65 years old. B: ≥ 65 years old.


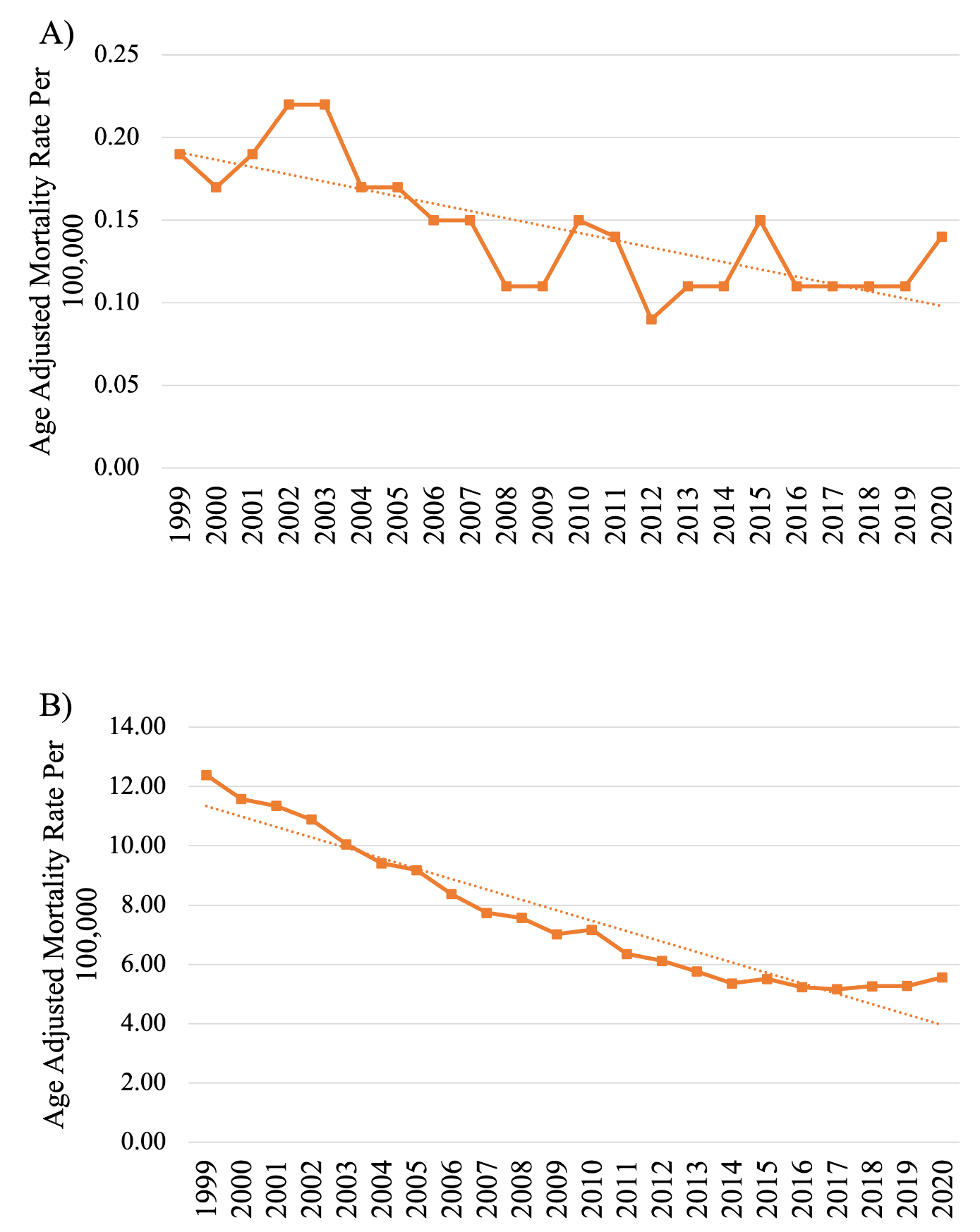


Supplementary Figure 2. Age‐Adjusted Mortality Rates of Cardiovascular Death among Patients with Comorbid Breast Cancer, Stratified by States.


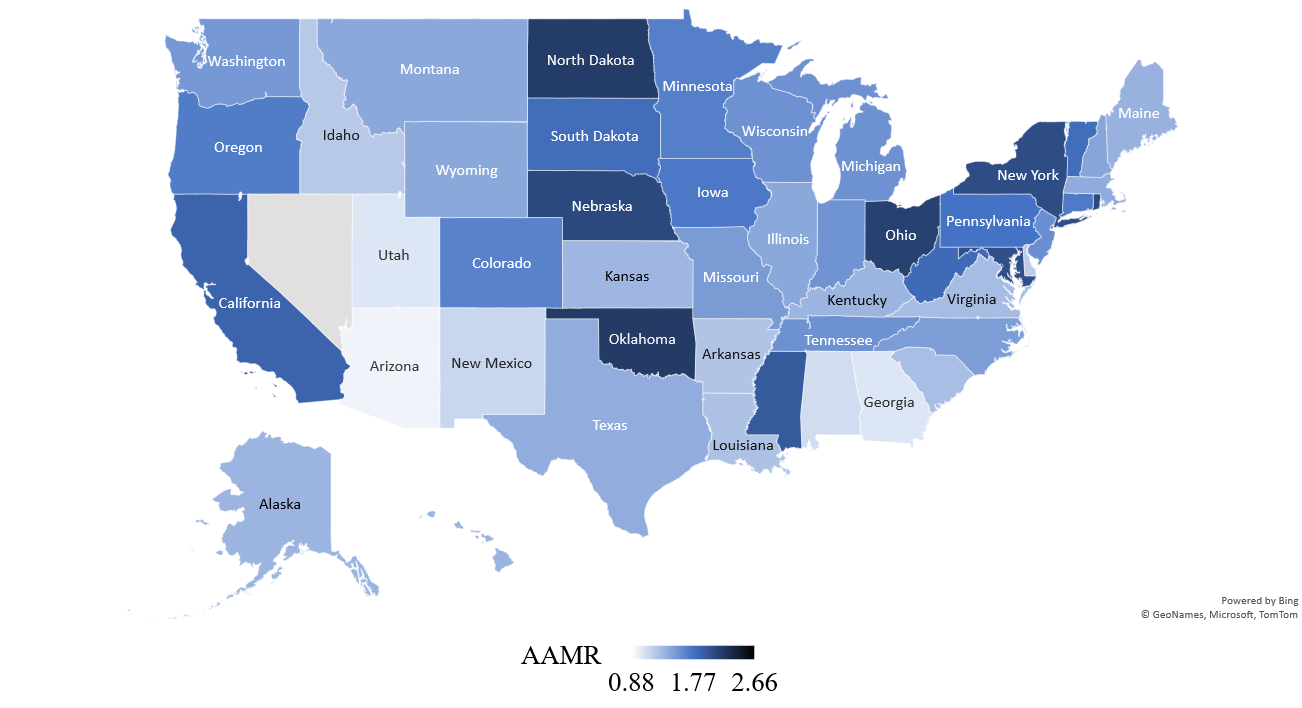

Supplement: Supplementary file 1 — Supplementary Material 1 [file 40959_2024_286_MOESM1_ESM.docx]
